# How to Meet the Last OIE Expert Surveillance Panel Recommendations on Equine Influenza (EI) Vaccine Composition: A Review of the Process Required for the Recombinant Canarypox-Based EI Vaccine

**DOI:** 10.3390/pathogens5040064

**Published:** 2016-11-25

**Authors:** Romain Paillot, Nicola L. Rash, Dion Garrett, Leah Prowse-Davis, Fernando Montesso, Ann Cullinane, Laurent Lemaitre, Jean-Christophe Thibault, Sonia Wittreck, Agnes Dancer

**Affiliations:** 1 Animal Health Trust, Lanwades Park, Kentford Newmarket CB8 7UU, UK; nicola.rash@aht.org.uk (N.L.R.); DION.GARRETT@aht.org.uk (D.G.); leah.prowse@aht.org.uk (L.P.-D.); fernando.montesso@aht.org.uk (F.M.); 2 Irish Equine Centre, Johnstown, Naas, W91 RH93 Co. Kildare, Ireland; ACullinane@irishequinecentre.ie; 3 Merial SAS, 69007 Lyon, France; Laurent.LEMAITRE@merial.com (L.L.); Jean-christophe.THIBAULT@merial.com (J.-C.T.); Sonia.WITTRECK@Merial.com (S.W.); Agnes.DANCER@merial.com (A.D.)

**Keywords:** horse, equine influenza, vaccination, canarypox, update

## Abstract

Vaccination is highly effective to prevent, control, and limit the impact of equine influenza (EI), a major respiratory disease of horses. However, EI vaccines should contain relevant equine influenza virus (EIV) strains for optimal protection. The OIE expert surveillance panel annually reviews EIV evolution and, since 2010, the use of Florida clade 1 and 2 sub-lineages representative vaccine strains is recommended. This report summarises the development process of a fully- updated recombinant canarypox-based EI vaccine in order to meet the last OIE recommendations, including the vaccine mode of action, production steps and schedule. The EI vaccine ProteqFlu contains 2 recombinant canarypox viruses expressing the haemagglutinin of the A/equine/Ohio/03 and A/equine/Richmond/1/07 isolates (Florida clade 1 and 2 sub-lineages, respectively). The updated EI vaccine was tested for efficacy against the representative Florida clade 2 EIV strain A/equine/Richmond/1/07 in the Welsh mountain pony model. Protective antibody response, clinical signs of disease and virus shedding were compared with unvaccinated control ponies. Significant protection was measured in vaccinated ponies, which supports the vaccine registration. The recombinant canarypox-based EI vaccine was the first fully updated EI vaccine available in the EU, which will help to minimise the increasing risk of vaccine breakdown due to constant EIV evolution through antigenic drift.

## 1. Introduction

**Figure 1:**
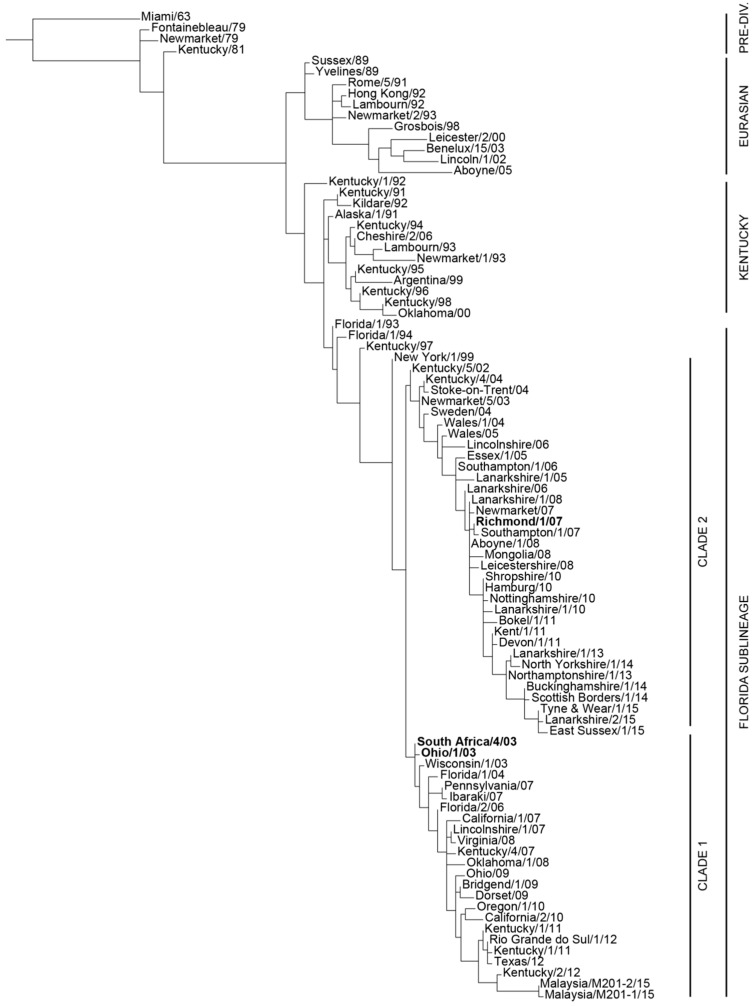
Phylogenetic analysis of the HA1 nucleotide sequences encoded by H3N8 equine influenza virus created using PhyML version 3. (ATGC: Montpellier Bioinformatics Platform; France) [1] Lineages and sub-lineages are indicated. The OIE ESP recommended strains A/equine/South Africa/4/03 (and closely related A/equine/Ohio/1/03) and A/equine/Richmond/1/07 are indicated in bold text. Courtesy of Dr. A. Rash, Equine Influenza Surveillance Programme (AHT/HBLB).

Equine Influenza (EI) is considered to be one of the most important respiratory diseases of horses, with welfare implications and the risk of substantial economic loss for the equine industry in the event of large outbreaks. Regretfully, this was particularly well illustrated during the 2007 Australian outbreak, when around 75,000 horses were affected and the overall cost reached A$1 billion. Equine influenza vaccination is widely recognised as a pivotal method of prevention. However, the equine influenza virus (EIV; the EI causative agent) constantly evolves in order to escape natural and/or vaccine immunities. Modification of EIV antigens (also called antigenic drift) may lead to a reduction in EI vaccine efficacy as a result of significant antigenic mismatch between the circulating EIV and the EI vaccine strains. In order to avoid EI vaccine breakdown and the associated risk of EI outbreaks, EIV antigenic evolution is closely monitored by the OIE Expert Surveillance Panel (OIE ESP) and associated Institutes and Laboratories. In this context, the OIE ESP provides annual recommendations on EI vaccine strain composition. Since 2010, incorporation of EIV representative strains from the Florida clade 1 and clade 2 sub-lineages (FC1 and FC2, respectively) is recommended (Figure 1). This report aims to briefly review and summarise the process required to update the recombinant canarypox-based EI vaccine in order to meet the last recommendation from the OIE ESP. Results from the pivotal study to confirm efficacy against the FC2 representative EIV strain A/equine/Richmond/1/07 are also presented.

## 2. Characteristics of the Recombinant Canarypox-Based EI Vaccine 

**Figure 2:**
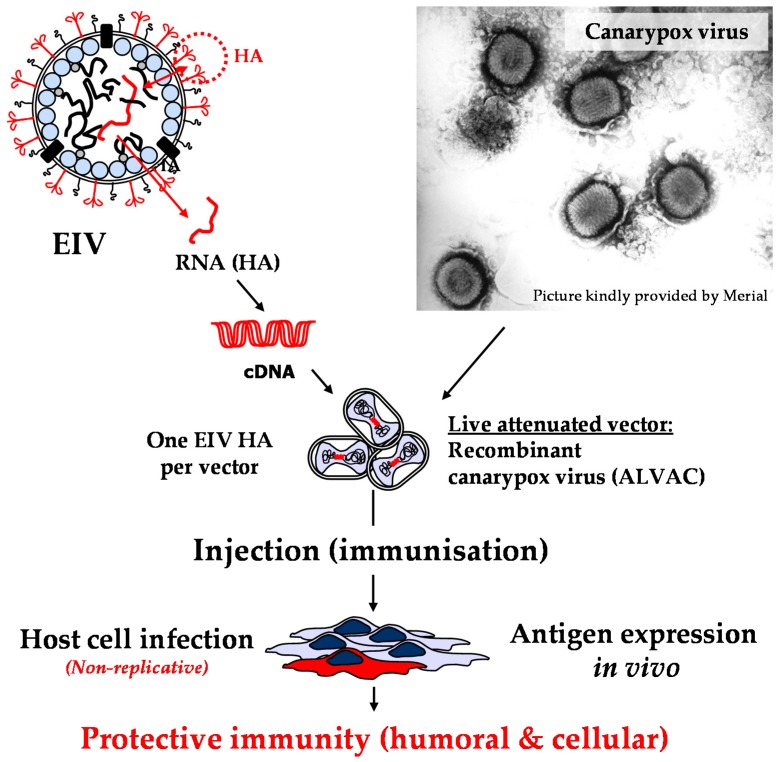
The canarypox vector (ALVAC) is a live attenuated virus with a large capacity to incorporate foreign genes. The equine influenza virus (EIV) (haemagglutinin) HA is the target antigen, the HA gene is inserted in the canarypox-vector genome (one EIV HA per vector). The EIV HA is expressed by host cells infected with the recombinant canarypox-vector after vaccine injection. Uptake and process of EIV HA will induce stimulation of an HA-specific protective immune response that involves both humoral and cell-mediated immunities [2]. The recombinant canarypox vector cannot multiply in mammalian cells (safety).

The parental canarypox is a double-stranded DNA virus from the Orthopoxvirus family that is host-specific and only replicates in avian species. The recombinant canarypox-based EI vaccine uses a replicative defective canarypox (ALVAC) vector (Figure 2). The ALVAC vector is derived from a single pox lesion on an infected canary. It is a single plaque isolate attenuated by 200 serial passages in chicken embryo fibroblast cell (CEF) culture and four successive plaque purifications. The ALVAC vector does not replicate in non-avian cells, which contributes to its safety in mammals.

The ALVAC vector has a large capacity to accept foreign genes, such as the EIV haemagglutinin (HA). The recombinant canarypox-based EI vaccine (i.e., ProteqFlu) currently commercialised (i.e., updated) contains two different recombinant ALVAC vectors expressing the HA (HA-ALVAC) of EIV strains A/equine/Ohio/03 (FC1 sub-lineage) and A/equine/Richmond/1/07 isolate (FC2 sub-lineage), respectively. After vaccine administration, equine cells are infected with the recombinant HA-ALVAC. However, infection is limited to the cytoplasm (the ALVAC vector never reaches the nucleus), which prevents potential risk of genetic recombination in case of co-infection with EIV (EIV ribonucleoproteins are imported to the nucleus were replication takes place). The HA protein is produced by the infected cells and presented to the immune system.

The recombinant canarypox-based EI vaccine stimulates both humoral and cell-mediated immunity (reviewed in [3,4]) and has a well-described efficacy against EIV infection. This EI vaccine is widely used, including in emergency situations such as the 2003 South African and 2007 Australian EI outbreaks [5,6]. Pre-existing immunity to the ALVAC vector does not interfere with further immunisation with the same or a different recombinant canarypox-based vaccine [3].

Differentiation of infected animals from vaccinates (DIVA) is essential for disease surveillance and outbreak management. As the canarypox-based EI vaccine contains solely the HA gene, horses immunised with this vaccine seroconvert against the EIV HA protein only. Detection of the immune response to other EIV structural proteins (e.g., nucleoprotein, NP) could be used to identify and differentiate infected from vaccinated horses [7,8]. The canarypox-based EI vaccine DIVA capacity was pivotal for its selection and use in Australia during the 2007 EI outbreak [6].

## 3. Updating the Recombinant Canarypox-Based EI Vaccine 

### 3.1. Design and Construction of the New ALVAC Vector

**Figure 3:**
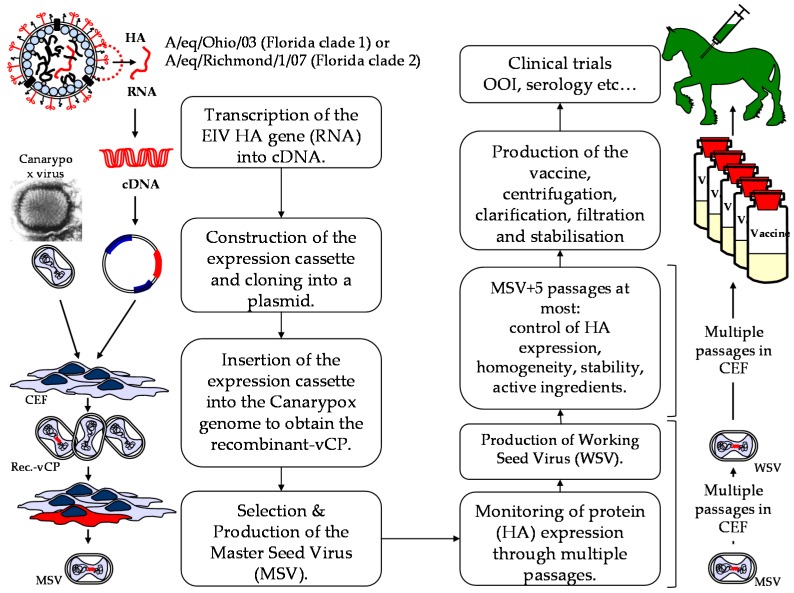
Summary of the methodological steps and process required to update the recombinant canarypox EI vaccine. OOI = onset of immunity.

Prior to 2014, the canarypox-based EI vaccine ProteqFlu contained two recombinant ALVAC vectors expressing the HA of EIV strains A/equine/Ohio/03 (Florida clade 1 sub-lineage) and A/equine/Newmarket/2/93 (European lineage), respectively. The EIV strain A/equine/Newmarket/2/93 was replaced with the A/equine/Richmond/1/07 isolate (Florida clade 2 sub-lineage) to meet the last OIE recommendations (Figure 3).

The HA gene from A/equine/Richmond/1/07 is a synthetic gene derived from the native sequence that is inserted in a donor plasmid (or expression cassette) between the C5R right flanking arm + H6 vaccinia virus promoter and the left flanking arm C5L. The recombinant ALVAC virus is obtained by in vitro homologous recombination after transfection of CEF with the donor plasmid and subsequent infection with the parental ALVAC vector. The recombinant ALVAC virus is plaque selected and amplified in CEF culture (positive for HA and negative for parental C5 ORF). HA expression is confirmed in recombinant ALVAC infected cell lysate by western blot using a monoclonal anti EIV HA antibody. The recombinant ALVAC virus obtained is defined as the master seed virus (MSV) [9].

The HA expression has been confirmed through multiple passages in CEF. The final vaccine construct was identified and titrated in CEF by HA-specific immunofluorescence. Global influenza antigen titres are expressed as FAID_50_ units (fluorescent assay infectious dose 50%). The two recombinant ALVAC vectors encode FC1 and FC2 EIV HA proteins of 565 and 567 amino acids, respectively, with 94% homology and 28 amino acids substitutions [10,11].

### 3.2. Validation, Batch Consistency and Stability

Validation of the different quantification, identification and control methods was also conducted and provided to the European Medicine Agency (EMA) (i.e., quantification of recombinant HA-ALVAC vectors contained in the vaccine by FAID_50_ and qPCR). The monoclonal antibody used to titrate the EIV HA A/equine/Ohio/03 (FC1 sublineage) cross-reacted with the EIV HA A/equine/Richmond/1/07 (FC2 sublineage) and, therefore, was used to confirm global HA expression.

The development of the new formulation has been based on the Committee for Veterinary Medicinal Products (CVMP) Note for Guidance on harmonisation of requirements for equine influenza vaccines, specific requirements for substitution or addition of a strain or strains (EMA/CVMP/112/98) [12] and has addressed all the requirements regarding production and quality controls (including batches consistency of the new active ingredient). Real-time stability studies were conducted with ALVAC vector titres monitored for 15 months at the time of registration and further completed up to 36 months. Results indicated that the loss of titre was similar to the loss described for the non-updated ProteqFlu vaccine. Therefore, the recommended shelf life of the vaccine remains unchanged compared to the previous formulation i.e., three years at 5 °C.

### 3.3. Safety Evaluation

The safety of the new recombinant canarypox-based EI vaccine (adjuvanted and with tetanus valency) was tested in accordance with the recommendations from the EMA CVMP [12]. Safety was tested in the natural host in three different conditions; when administered as an overdose, a single dose injection and repeated dose administration in foals and when compared to the non-updated ProteqFlu-TE. Typical systemic and site reactions were recorded after vaccine administration (i.e., slight pyrexia and transient local inflammation). Interestingly, safety of the new ALVAC vector expressing EIV A/equine/Richmond/1/07 HA was also tested in canaries. The new ALVAC construct and the bare parental ALVAC vector are safe, with an absence of intra-host dissemination, shedding and transmission to sentinel canaries when administered at a high dose by the transcutaneous route (data not shown).

## 4. Updated Recombinant EI Vaccine: Efficacy Results against a Florida Clade 2 EIV Strain

The updated recombinant canarypox-based EI vaccine was tested for efficacy against the representative FC2 EIV strain A/equine/Richmond/1/07 in the Welsh mountain pony model. Seven seronegative Welsh mountain ponies were vaccinated at minimum protective dose (1/100 commercial release dose) twice (V1 andV2, five weeks apart) with the updated recombinant canarypox-based EI vaccine, prior to experimental infection with the EIV A/equine/Richmond/1/07 (two weeks after V2). Protective antibody response, clinical signs of disease and virus shedding were compared with unvaccinated control ponies (*n* = 7).

### 4.1. Serological Response

**Figure 4:**
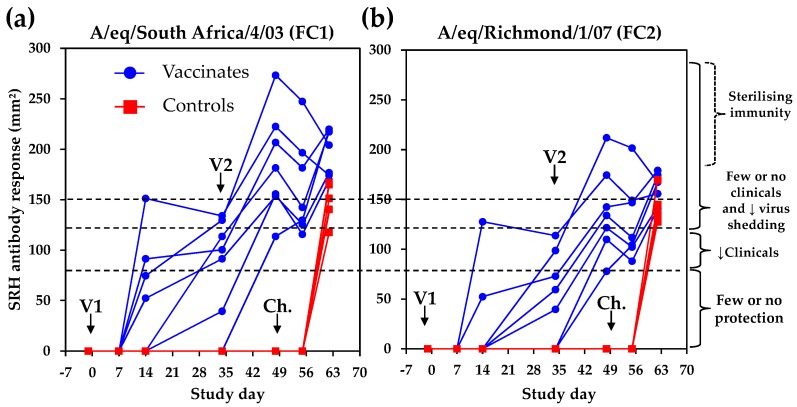
SRH antibody response to A/equine/South Africa/4/03 (**a**) or A/equine/Richmond/1/07; (**b**) after vaccination and experimental infection with A/equine/Richmond/1/07. SRH antibody protection thresholds are indicated. Clinicals = clinical signs of disease.

The single radial haemolysis (SRH) antibody response was measured against the EIV strains A/equine/South Africa/4/03 (FC1) and A/equine/Richmond/1/07 (FC2). As illustrated in Figure 4, six out of seven vaccinates seroconverted against at least one antigen after V1 (average SRH antibody values on Day 34 were 86.9 ± 49.6 mm^2^ and 54.9 ± 44.7 mm^2^ against FC1 and FC2 EIV strains, respectively). All vaccinates were seropositive after V2. On Day 48 and prior to experimental infection (Day 49), SRH values were 186.7 ± 52.6 mm^2^ and 138.8 ± 43.8 mm^2^ against FC1 and FC2 EIV strains, respectively. All of the control ponies remained seronegative prior to experimental infection. The kinetics of the SRH antibody response was confirmed using the FC2 EIV strain A/equine/Meath/1/07 (data not shown).

### 4.2. Clinical Protection

**Figure 5:**
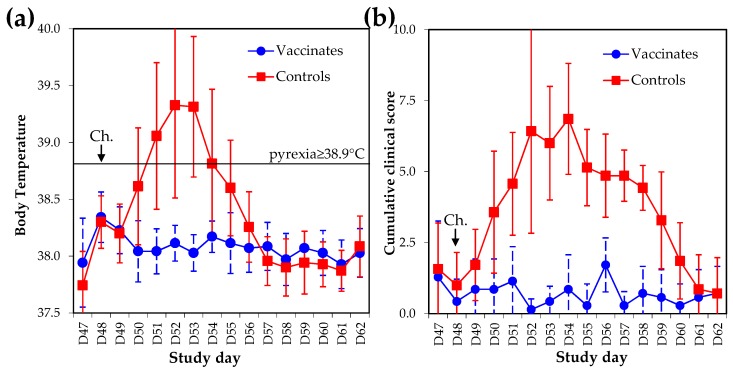
Clinical disease. (**a**) Body temperature after experimental infection with EIV A/equine/Richmond/1/07. Body temperature ≥38.9 °C is considered pyretic (horizontal line); (**b**) cumulative score of disease. Ch. = challenge with EIV A/equine/Richmond/1/07.

The severity of clinical signs of disease (i.e., cumulative clinical score/duration) induced by experimental infection with EIV A/equine/Richmond/1/07 were statistically reduced in vaccinated ponies when compared with controls ponies (*p* < 2.1×10^−7^, Student’s *t*-test) (Figure 5). All but one control pony developed pyrexia (i.e., body temperature ≥38.9 °C) for 3.1 ± 2.1 days on average. None of the vaccinates were pyretic (*p* < 0.005, Wilcoxon test). Duration of coughing and nasal discharge were also statistically reduced in vaccinates (*p* < 0.002, Wilcoxon test and *p* < 0.001, Student’s *t*-test, respectively). All control ponies required antibiotic treatment (Trimethoprim and Sulfadiazine) for 5–7 days to support recovery (treatment was initiated from day 5 to day 10 post experimental infection). No vaccinates required treatment (*p* < 0.0006, Fisher’s Exact Test).

### 4.3. Virological Protection

**Figure 6:**
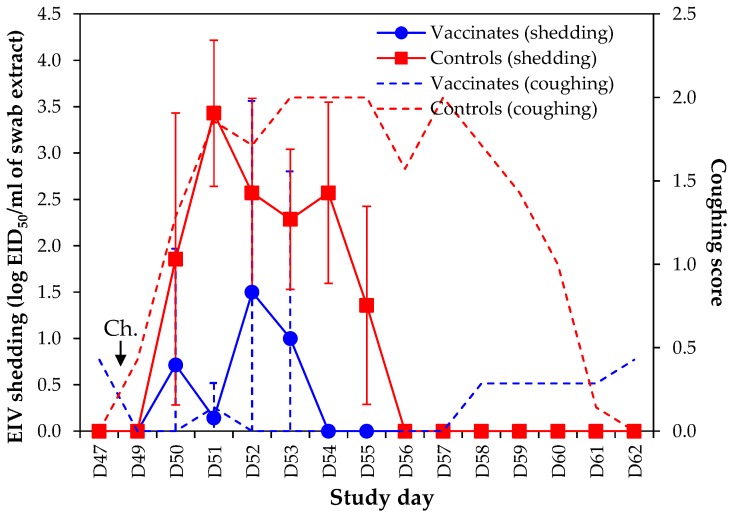
EIV shedding measured by titration in embryonated hens’ eggs and coughing score. Ch. = challenge with EIV A/equine/Richmond/1/07.

Equine influenza virus shedding was significantly reduced in vaccinated ponies when compared with control ponies (Figure 6). Three out of seven vaccinated ponies had detectable EIV in nasal swab samples, while all controls ponies were positive. A strict analysis of EIV shedding indicated that three vaccinated ponies were positive for 2–3 days, when compared to 5–6 days for control ponies (duration of EIV shedding was 1.1 ± 1.5 days in vaccinates and 5.4 ± 0.5 days in control ponies; *p* < 0.00001, Student’s *t*-test). Overall duration of virus shedding (i.e., from first positive day to last positive day, even in case of intermittent virus shedding) was 1.4 ± 1.8 days in vaccinated ponies (3–4 days duration) when compared to 5.4 ± 0.5 days in control ponies (5–6 days duration; *p* < 0.001, Student’s *t*-test). Overall cumulative EIV titres were also statistically significantly reduced (*p* < 0.001, Student’s *t*-test). The significant decrease in EIV shedding measured in vaccinated ponies was also confirmed by EIV NP qRT-PCR (Supplementary data #1).

### 4.4. Potential Sterilising Immunity to EI

**Figure 7:**
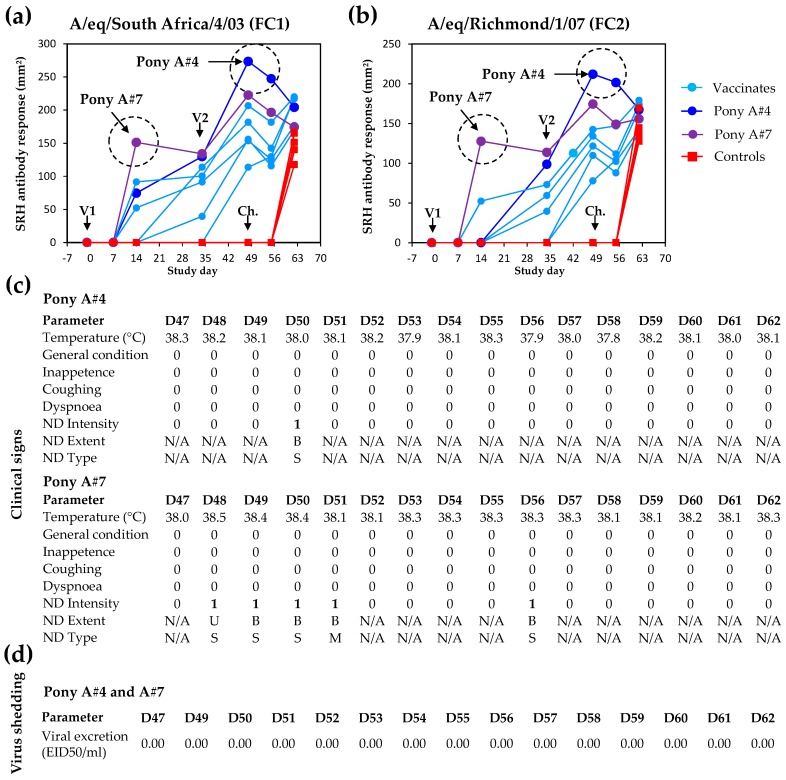
Potential example of vaccine-induced sterilising immunity (Pony A#4 and A#7). SRH antibody response against A/equine/South Africa/4/03 (**a**) or A/equine/Richmond/1/07 (**b**); Pony A#4 and A#7 individual clinical signs of disease (**c**). Virus shedding was measured by titration in embryonated hens’ eggs (**d**). Positive clinical signs or virus shedding are indicated in bold text. ND = nasal discharge, ND extent U = unilateral and B = bilateral, ND type S = serous and M = mucopurulent.

The absence of EI-related clinical signs, virus shedding, and seroconversion is usually considered a markers of sterile immunity to EIV infection, which is rarely achieved. Two vaccinated ponies A#4 and A#7 showed the highest SRH antibody values measured against both antigens at the time of challenge (Figure 7). Most vaccinated ponies showed a typical decrease of SRH antibody values after V2 (i.e., illustrated here between Day 49 and Day 55) [13], followed by a significant increase induced by experimental infection with EIV (i.e., acting as immunological boost) and measured between Day 55 and Day 62. However, ponies A#4 and A#7 did not seroconvert after experimental infection with EIV A/equine/Richmond/1/07, and displayed few clinical signs of disease (limited to non-specific mild nasal discharge, which may be unrelated to EIV infection), and no infectious virus shedding (Figure 7). Such serological response, clinical and virological protection observed after experimental infection with EIV support the possibility of sterilising immunity for ponies A#4 and A#7 (i.e., negligible or no infection despite infectious EIV challenge).

### 4.5. Other Efficacy Tests

Bioequivalence to the non-updated ProteqFlu-TE was demonstrated in foals during five months after the primo-vaccination. Twenty young horses (13–28 months of age) were vaccinated with the new recombinant canarypox-based EI vaccine (adjuvanted and with tetanus valency; *n* = 10) or the non-updated ProteqFlu-Te (*n* = 10). Two foals were kept as unvaccinated controls. Foals received three injections on day 0 (V1), day 35 (V2), and day 189 (V3). SRH antibody responses were measured against the EIV strains A/equine/South Africa/4/03 (FC1 sub-lineage), A/equine/Meath/1/07 (homologous to A/equine/Richmond/1/07, FC2 sub-lineage), and A/equine/Newmarket/2/93 (European lineage). SRH antibody values measured between groups were found to be equivalent between groups (data not shown) [10,11].

Efficacy of the new recombinant canarypox-based EI vaccine was not tested in vivo against EIV strains from the FC1 sub-lineages. The non-updated ProteqFlu vaccine already contained a recombinant ALVAC vector expressing the HA of the EIV strain A/equine/Ohio/03 and efficacy against an homologous challenge strain had been previously demonstrated and presented to the vaccine registration authorities [10].

## 5. Discussion

Results from the clinical studies clearly indicate that immunisation with the updated recombinant canarypox-based EI vaccines was safe and significantly reduced the severity/duration of disease and the intensity/duration of virus shedding induced by experimental infection with the FC2 EIV strain A/equine/Richmond/1/07, 2 weeks after the second vaccination. Significant protection against EIV infection and subsequent disease is expected from EI vaccines when tested at the onset of immunity (i.e., shortly after boost immunisation). However, sterilising immunity is usually difficult to achieve. Evidences of such extended protection were measured for two of the vaccinated ponies at a minimum protective dose (1/100 commercial release dose).

Antimicrobial resistance is considered to be one of the most important threats and challenges to human and animal health in the next decades. The beneficial impact of EI vaccination on the occurrence of secondary bacterial infections is very clearly illustrated here, with no antibiotic treatment necessary in the group of vaccinated ponies.

The lack of specific reagents, such as a FC2 European Pharmacopoeia reference serum standard, complicates and slows the updating process. A common OIE International Standard/European Pharmacopoeia Biological Reference Preparation (BRP; reference serum standard) to the FC2 EIV strain A/equine/Richmond/1/07 is currently being validated [14].

Irrespective of the EI vaccine type and nature, meeting the OIE ESP recommendation on strain composition is a lengthy process. New vaccine technologies [3] may also provide an alternative way to achieve this process in the future.

## 6. Materials and Methods

### 6.1. Experimental Animals and Vaccination Protocol

Eligibility criteria: all animals (approximately 10 months of age, males and females) were in good health and seronegative for EIV, with no history of exposure to EIV, and no history of vaccination against EIV or with a canarypox-based vaccine at the time of the studies.

#### 6.1.1. Setting, Location, and Sample Size

Ponies were enrolled by the study investigator. Fourteen Welsh mountain ponies were used and housed together on Animal Health Trust (AHT) premises. The day before experimental infection with EIV, ponies were transferred to a category II containment facility. Ponies were released 14 days after experimental infection. Sample size was based on power calculations and to meet the European Pharmacopoeia criteria for equine influenza vaccine (inactivated), with no fewer than six and four horses for the treated and control groups, respectively [15].

#### 6.1.2. Investigational Veterinary Product (IVP) and Intervention

Recombinant canarypox vaccine in liquid formulation containing vCP2242 (expressing HA from A/equine/Ohio/03), vCP3011 (expressing HA from A/equine/Richmond/1/07) and tetanus toxoid (100 Lf/mL) in Carbomer adjuvant (4 mg/mL). The global influenza antigen titre was 5.6 log_10_ FAID_50_/mL. (1:1 ratio, with 5.3 log_10_ FAID_50_/mL for each vCPs). The vaccine was administered by deep intramuscular injection (21G × 1½”, 0.8 × 40 mm needle) in the left neck (one dose) on Day 0 and Day 34. Control ponies were kept unvaccinated.

#### 6.1.3. Randomisation and Masking

The 14 ponies included in the study were assigned to two groups of seven animals each, by randomisation based on sex and identification number using a four-element permutation table. Personnel at the study site responsible for performing clinical and general health observations or involved in laboratory assays were not informed about the allocation of individual animals at any time during the study.

#### 6.1.4. Animal Welfare

All experiments involving animals presented here were carried out in compliance with UK legislation and were subjected to ethical review (AHT and Merial SAS). This report of clinical trials follows the CONSORT 2010 guidelines (supplementary CONSORT check list and flowchart) [16,17].

### 6.2. Viruses and Experimental Infection with EIV

Viruses were all grown in embryonated hen’s eggs, purified, and titrated as described previously [18]. The EIV strain A/equine/Richmond/1/07 (passage 5 in eggs) was used for experimental infection and was titrated in embryonated hens’ eggs prior to challenge (day 48). The titre was defined using the method of Reed and Muench and expressed in EID_50_ per mL [19]. The ponies were divided into two groups of seven (three controls + four vaccinates in the first room; four controls + three vaccinates in the second room) and experimentally infected by room nebulisation of an infectious EIV suspension containing a total of 10^7.6^ EID_50_ EIV strain A/equine/Richmond/1/07 (ULTRA 2000 nebuliser, DEVibiss, Somerset, PA, USA) per rooms as described previously [20].

### 6.3. Clinical Signs of Disease (Pre-Specified Analysis)

#### Outcome Measure

Clinical examinations were performed daily on each pony from day 47 (one day prior to challenge) until day 62 (challenge + 14 days) for the occurrence of clinical signs associated with EI as previously described [20,21]. Rectal temperatures greater than 38.8 °C were regarded as pyretic. The sickness score after experimental infection with EIV was calculated using the daily score for each clinical sign according to the formula previously reported [21]: sickness score = (2 × score RT) + score nasal discharge + score cough + 2 × (score dyspnoea + anorexia + depression). This calculation was performed by the AHT to be consistent with previously published studies. Ponies developing/cumulating long lasting (>3 days) and moderate clinical signs after experimental infection with EIV were treated to control secondary bacterial infection and improve recovery, as per the AHT ethical guidelines.

### 6.4. Virus Shedding

#### Outcome Measure

nasopharyngeal swabs were taken from each pony on day 47 and daily for 14 days from day 49 to day 62, excluding day 48 (challenge infection). Swabs were processed in 5 mL of virus transport medium (PBS, 200 U/mL streptomycin, 150 U/mL penicillin, 5 mg/mL amphotericin B and 600 mg/mL tryptone phosphate broth, all supplied by Sigma-Aldrich Co. Ltd, Gillingham, Dorset, UK) and stored around −70 °C prior to analysis by embryonated hens’ egg titration [22]. The titration in embryonated eggs assesses the presence of live infectious virus. Results are expressed as log EID_50_/mL [19,20] of swab extract as previously described [21]. Two sets of statistical analysis of EIV were conducted. A “strict” analysis based on number of days positive for EIV shedding and an “overall duration” of virus shedding based on periods of shedding defined from the first positive day to the last positive day. Overall duration is also measured due to intermittent virus excretion which is potentially a result of alternative nostrils being sampled each day (e.g., EIV shedding measured on days 3, 4, and 6 was defined as a four-day period).

### 6.5. Serology

#### 6.5.1. Outcome Measure (Pre-Specified Analysis)

Serum samples were collected on day −1, day 7, day 14, day 34, day 48 (experimental infection), day 55, and day 62, and analysed by SRH assays [22] against the EIV strains A/equine/South Africa/4/03 (FC1 sublineage) and A/equine/Richmond/1/07 (FC2 sublineage) used as antigens. SRH antibody values were expressed as the area of haemolysis (mm^2^). An increase of at least 25 mm^2^ or 50% in the area of the zone of haemolysis was regarded as significant. European Pharmacopoeia reference serum standard (BRP) Eu SA/4/03 Y0000712 (A/equine2/South Africa/4/2003) was used as a positive control sera (data not shown). 

#### 6.5.2. Exploratory Analysis

Serum samples were also analysed by SRH assay against the FC2 EIV strain A/equine/Meath/1/07. An in-house reference serum standardised with the European Pharmacopoeia serum standard against A/equine/Newmarket/1/93 strain was used as a positive control.

### 6.6. Statistical Analysis

Statistical analyses were performed with STATGRAPHICS Centurion XVI, version 16.1.12 (StatPoint Technologies, Inc., Warrenton, VA, USA). Where appropriate, based on the normality test for group distribution, Student’s *t*-test (S) or Wilcoxon signed rank test (W) were used to compare groups at specific time points. A two-tailed Fisher’s Exact Test was used to compare treatment occurrence per group. The level of significance was set as *p* < 0.05.

## 7. Conclusions

**Figure 8:**
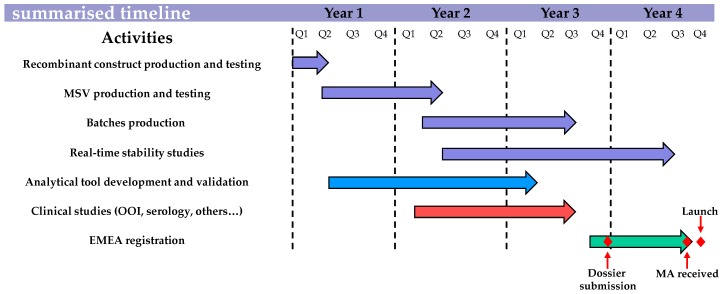
Summarised timeline for the recombinant canarypox-based EI vaccine update.

Despite EMA adapting its requirements, updating the canarypox-based EI vaccine in order to meet the last OIE ESP recommendations on EI vaccine strain composition and the EMA quality, safety, and efficacy requirements was a complex four year process, as illustrated and summarised in Figure 8. The recombinant canarypox-based EI vaccine was the first fully-updated EI vaccine available in the EU, which will help to minimise the increasing risk of vaccine breakdown due to constant EIV evolution through antigenic drift.

## Supplementary Materials

The following are available online at www.mdpi.com/2076-0817/5/4/64/s1, Figure S1: CONSORT flow diagram, Table S1: CONSORT check list. Supplementary data S1: measure of EIV shedding by EIV NP RT-PCR.

## Abbreviations

The following abbreviations are used in this manuscript:

AHT	Animal Health Trust
ALVAC	replicative defective canarypox vector
CMI	Cell-Mediated Immunity
CEF	Chicken Embryo Fibroblasts
CVMP	Committee for Veterinary Medicinal Products
EI	Equine Influenza
EIV	Equine Influenza Virus
EMA	European Medicine Agency (formerly known from 1995 to 2004 as EMEA, European Agency for the Evaluation of Medicinal Products)
FC1 and FC2	Florida Clade 1 and Florida Clade 2, respectively
HA	Haemagglutinin
HBLB	Horserace Betting Levy Board
IVP	Investigational Veterinary Product
MDPI	Multidisciplinary Digital Publishing Institute
MSV	Master Seed Virus
WSV	Working Seed Virus

## References

[1] Guindon S., Gascuel O. (2003). A simple, fast, and accurate algorithm to estimate large phylogenies by maximum likelihood. Syst. Biol..

[2] Paillot R., Kydd J.H., Sindle T., Hannant D., Edlund Toulemonde C., Audonnet J.C., Minke J.M., Daly J.M. (2006). Antibody and IFN-gamma responses induced by a recombinant canarypox vaccine and challenge infection with equine influenza virus. Vet. Immunol. Immunopathol..

[3] Paillot R. (2014). A systematic review of recent advances in equine influenza vaccination. Vaccines.

[4] Paillot R., Hannant D., Kydd J.H., Daly J.M. (2006). Vaccination against equine influenza: Quid novi?. Vaccine.

[5] Guthrie A.J. (2006). Equine Influenza in South Africa 2003 Outbreak. 9th International Congress of World Equine Veterinary Association, Marrakech, Morocco, 22–26 January 2006.

[6] Paillot R., El-Hage C.M. (2016). The use of a recombinant canarypox-based equine influenza vaccine during the 2007 Australian outbreak: A systematic review and summary. Pathogens.

[7] Kirkland P.D., Delbridge G. (2011). Use of a blocking elisa for antibodies to equine influenza virus as a test to distinguish between naturally infected and vaccinated horses: Proof of concept studies. Aust. Vet. J..

[8] Galvin P., Gildea S., Arkins S., Walsh C., Cullinane A. (2013). The evaluation of a nucleoprotein ELISA for the detection of equine influenza antibodies and the differentiation of infected from vaccinated horses (diva). Influenza Other Respir. Viruses.

[9] European Medicines Agency Proteqflu: Epar-Scientific Discussion. http://www.ema.europa.eu/docs/en_GB/document_library/EPAR_-_Scientific_Discussion/veterinary/000073/WC500065184.pdf.

[10] European Medicines Agency (2014). Committee for Medicinal Products for Veterinary Use Cvmp Assessment Report for Type II Variation for Proteqflu (Emea/v/c/000073/ii/0014).

[11] European Medicines Agency (2014). Committee for Medicinal Products for Veterinary Use Cvmp Assessment Report for Type II Variation for Proteqflu te (Emea/v/c/000074/ii/0017).

[12] European Agency for the Evaluation of Medicinal Products (1998). Committee for Veterinary Medicinal Products, Harmonisation of Requirements for Equine Influenza Vaccines: Specific Requirements for Substitution or Addition of a Strain or Strains. Emea/Cvmp/112–98-Final.

[13] Gildea S., Arkins S., Walsh C., Cullinane A. (2011). A comparison of antibody responses to commercial equine influenza vaccines following primary vaccination of thoroughbred weanlings—A randomised blind study. Vaccine.

[14] Paillot R., Lopez-Alvarez M.R., Garrett D., Birand I., Behr-Gross M.-E. (2016). Production and establishment of a new candidate horse antiserum (common oie international standard/european pharmacopoiia biological reference preparation) to the florida clade 2 equine influenza virus a/eq/richmond/1/07. J. Equine Vet. Sci..

[15] (2010). Equine Influenza Vaccine (Inactivated). European Pharmacopoeia.

[16] Moher D., Hopewell S., Schulz K.F., Montori V., Gotzsche P.C., Devereaux P.J., Elbourne D., Egger M., Altman D.G. (2010). Consort 2010 explanation and elaboration: Updated guidelines for reporting parallel group randomised trials. BMJ.

[17] Schulz K.F., Altman D.G., Moher D. (2010). Consort 2010 statement: Updated guidelines for reporting parallel group randomised trials. PLoS Med..

[18] Paillot R., Grimmett H., Elton D., Daly J.M. (2008). Protection, systemic IFN and antibody responses induced by an iscom-based vaccine against a recent equine influenza virus in its natural host. Vet. Res..

[19] Reed L.J., Muench H. (1938). A simple method of estimating fifty percente endpoints. Am. J. Hyg..

[20] Paillot R., Prowse L., Montesso F., Huang C.M., Barnes H., Escala J. (2013). Whole inactivated equine influenza vaccine: Efficacy against a representative clade 2 equine influenza virus, IFNgamma synthesis and duration of humoral immunity. Vet. Microbiol..

[21] Paillot R., Prowse L., Donald C., Medcalf E., Montesso F., Bryant N., Watson J., Jeggo M., Elton D., Newton R. (2010). Efficacy of a whole inactivated EI vaccine against a recent EIV outbreak isolate and comparative detection of virus shedding. Vet. Immunol. Immunopathol..

[22] OIE Equine Influenza (Infection with Equine Influenza Virus): Chapter 2.5.7. http://www.oie.int/fileadmin/Home/eng/Health_standards/tahm/2.05.07_EQ_INF.pdf.

