# Supplementary material for: How to Meet the Last OIE Expert Surveillance Panel Recommendations on Equine Influenza (EI) Vaccine Composition: A Review of the Process Required for the Recombinant Canarypox-Based EI Vaccine
Source: Pathogens. 2016 Nov 25;5(4):64. doi: 10.3390/pathogens5040064 (PMC5198164; doi:10.3390/pathogens5040064)
Supplement: Supplementary file 1 [file pathogens-05-00064-s001.zip › Paillot et al Pathogens CONSORT flow diagram SEP16.pdf]

CONSORT

TRANSPARENT REPORTING of TRIALS

CONSORT 2010 Flow Diagram: How to meet the last OIE expert surveillance panel recommendations on Equine Influenza (EI) vaccine composition? A review of the process required for the recombinant canarypox-based EI vaccine.

Enrollment

Assessed for eligibility (n=14)

Excluded (n=0)
- Not meeting inclusion criteria (n=0)
- Declined to participate (n=0)
- Other reasons (n=0)

Randomized (n=14)

Allocation

Allocated to intervention (n=7)
Control: no product
- Received allocated intervention (n=7)
- Did not receive allocated intervention (give reasons) (n=0)

Allocated to intervention (n=7)
Vaccinates: IVP.
- Received allocated intervention (n=7)
- Did not receive allocated intervention (give reasons) (n=0)

Follow-Up

Lost to follow-up (give reasons) (n=0)

Discontinued intervention (give reasons) (n=0)

Lost to follow-up (give reasons) (n=0)

Discontinued intervention (give reasons) (n=0)

Analysis

Analysed (n=7)
- Excluded from analysis (give reasons) (n=0)

Analysed (n=7)
- Excluded from analysis (give reasons) (n=0)
