# Supplementary material for: How to Meet the Last OIE Expert Surveillance Panel Recommendations on Equine Influenza (EI) Vaccine Composition: A Review of the Process Required for the Recombinant Canarypox-Based EI Vaccine
Source: Pathogens. 2016 Nov 25;5(4):64. doi: 10.3390/pathogens5040064 (PMC5198164; doi:10.3390/pathogens5040064)
Supplement: Supplementary file 1 [file pathogens-05-00064-s001.zip › Paillot et al EIV Proteq Pathogens 2016 Supplementary data #1 SEP16.pdf]

How to meet the last OIE expert surveillance panel recommendations on Equine Influenza (EI) vaccine composition? A review of the process required for the recombinant canarypox-based EI vaccine.

Authors: Romain Paillot, Nicola Rash, Dion Garrett, Leah Prowse-Davis, Fernando Montesso, Laurent Lemaitre, Jean-Christophe Thibault, Sonia Wittreck and Agnes Dancer.

Supplementary Data #1: measure of EIV shedding by EIV NP RT-PCR

Methods: nasopharyngeal swabs were taken from each pony on day 47 and daily for 14 days from day 49 to day 62, excluding day 48 (challenge infection). Swabs were processed in 5ml of virus transport medium (PBS, 200U/ml streptomycin, 150U/mL penicillin, 5mg/ml amphotericin B and 600mg/ml tryptone phosphate broth, all supplied by Sigma-Aldrich Co) and stored around -70°C prior to analysis by EIV nucleoprotein reverse transcriptase polymerase chain reaction (EIV NP RT-PCR). The EIV NP qRT-PCR measures the presence of genetic material. The EIV NP quantitative RT-PCR results were expressed as number of EIV NP RNA copy per 2µl of swab extract, positive results ≥100 or ≥2 if log transformed. The 100 cut-off has been determined by comparison with the EIV NP ELISA, which was used as gold standard diagnostic method prior to the qRT-PCR assay development. The sensitivity and specificity of the qRT-PCR are 95.8% and 89.1%, respectively, with a cut-off threshold of 100 mRNA copies. The sensitivity of the assay has been favoured to maximise the surveillance for a relatively rare infection among diagnostic sample throughput. The specificity of the qRT-PCR is likely to be under-evaluated, which is not unusual when the new assay is superior to the gold standard method in place against which it is being compared [17]. Detection of EIV shedding by qRT-PCR is referred to in the OIE Terrestrial Manual EIV (2016) but no EIV qRT-PCR methods have been validated in line with the OIE validation template yet [19].

Results: Equine influenza virus shedding was significantly reduced in vaccinated ponies when compared with control ponies (Figure 1). Three out of seven vaccinated ponies had detectable EIV in nasal swab samples for 3 to 4 days while all controls ponies had detectable EIV for 5 to 6 days. Duration of EIV shedding measured by qRT-PCR was 2.7±2.3 days in vaccinates and 5.9±2.4 days in control ponies (p-value<0.0005; Student's t-test). Overall cumulative EIV titres were also statistically significantly reduced (p-values<0.0001, Student's t-test).

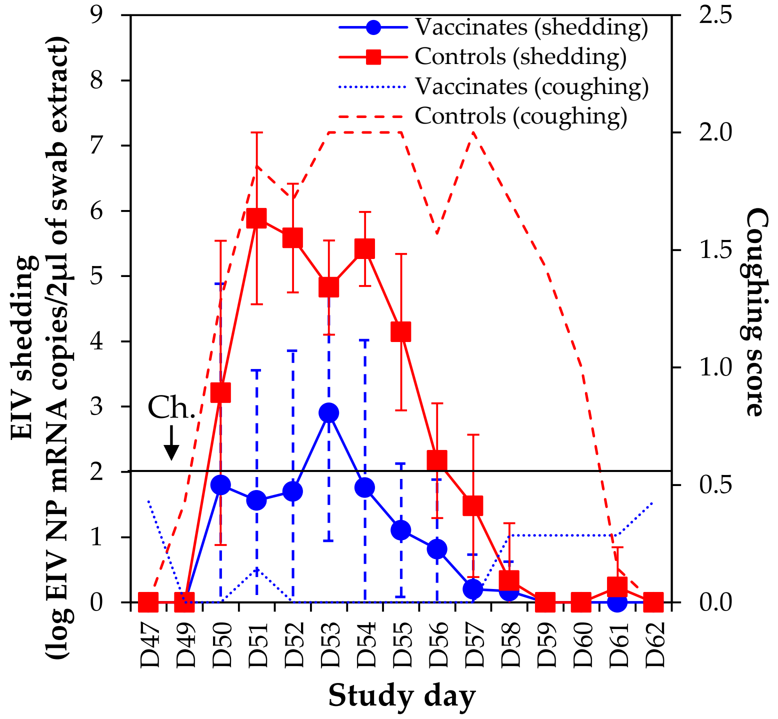

Figure 1. EIV shedding and coughing score. EIV shedding measured by qRT-PCR and coughing score. Titers ≥ 2 log EIV NP mRNA copies/2µl of nasopharyngeal swab extract are considered positive (horizontal line). (Ch.=challenge with EIV A/equine/Richmond/1/07.
